# Morphological patterns of lip prints in an Iranian population

**DOI:** 10.4317/jced.52921

**Published:** 2016-12-01

**Authors:** Mahkameh Moshfeghi, Amirreza Beglou, Hamed Mortazavi, Nazanin Bahrololumi

**Affiliations:** 1 Associate Professor, Department of Oral and Maxillofacial Radiology, Dental School, Shahid Beheshti University of Medical Sciences, Tehran, Iran; 2 Dentist, Dental School, Shahid Beheshti University of Medical Sciences, Tehran, Iran; 3 Associate Professor, Department of Oral Medicine, Dental School, Shahid Beheshti University of Medical Sciences, Tehran, Iran

## Abstract

**Background:**

Lip prints are verified to be unique to an individual and stable over time; hence they have potential for human identification purposes. The aim of this study was to assess the individuality and variability of lip prints in an Iranian population for the first time. We also sought to assess the possibility of sex determination via lip printing.

**Material and Methods:**

Lip prints of 96 individuals including 22 males and 74 females were recorded on a plain white paper using a dark-colored lipstick and 50 mm of Scotch tape. Each lip print was divided into six sextants and studied independently by two observers using a magnifying lens to examine the lip grooves. The Suzuki and Tsuchihashi’s classification was used to define the lip patterns and the data were statistically analyzed.

**Results:**

In the present study, no identically similar lip prints were observed. Type V was the most predominant pattern recorded in the study sample (33.16%), followed by type I (24.13%), type II (18.75%), type IV (11.63%), type I’ (9.72%) and type III (2.60%). In addition, no statistically significant difference was observed in the lip print patterns of males and females.

**Conclusions:**

It can be concluded that lip prints are unique and their analysis may enable human identification.

** Key words:**Forensic anthropology, forensic dentistry, forensic medicine, iran, lip.

## Introduction

Human identification plays an important role in criminal investigations and forensic medicine (1,2). Fingerprints, DNA testing, and dental records are conventional methods used as cornerstones in this context, allowing fast and reliable identification (3,4). However, under certain circumstances the use of the aforesaid methods is not possible (5). Thus, there is still an increasing need for adjunct techniques such as assessment of lip prints and palatal rugae patterns (6).

Lip print refers to the imprint produced by the natural lines and wrinkles in the vermilion zone of the lips (7,8). The study of the lip prints is known as cheiloscopy (2,4). According to Caldas (4), the term cheiloscopy was first coined in 1902. Santos for the first time suggested a classification for the lip print followed by others including Renaud (9); however, the classification by Suzuki and Tsuchihashi seems to be the most widely accepted classification (10). Suzuki and Tsuchihashi reported that although there are similarities between the lip prints of uniovular twins, they are not exactly identical (10).

Lip prints can be recognized as early as the sixth week of intrauterine life (5). Once formed, they do not change during an individual’s lifetime (6,11). However, resisting environmental factors such as minor trauma, inflammation, herpetic lesions and major or repeated trauma may alter the pattern and morphology of the wrinkles and flaw cheiloscopic studies (12,13).

Lip prints are verified to be unique to an individual and stable over time and, hence have the potential for human identification purposes (7,8). They can be obtained from various objects such as clothing, cups, glasses, cigarettes, and duct tapes (such as those used to bind a victim) (5,14). Chieloscopy is not only about studying visible prints but also the latent ones, made by the moisture provided by minor salivary and sebaceous glands of the lips, which can be rendered visible by substances like aluminum or silver powder (15,16).

In addition, similarities have been noted between the lip prints of parents, children, and siblings. Many studies have suggested the possibility of presence of gender differences in lip prints (7,14).

Despite the significant role of lip printing in distinguishing individuals and its common presence in crime scenes, studies in this regard are scarce. Lack of comprehensive databases of lip prints restricts unanimous acceptance of lip printing (5,7).

The aim of this study was to assess the variations in lip patterns of an Iranian population for the first time and evaluate the differences between sexes in this respect. In addition, we sought to determine the reliability and validity of lip prints.

## Material and Methods

-Subjects

This cross-sectional study carried out in April and July of 2015 was conducted on 158 patients referred to the Dental School of Shahid Beheshti University of Medical Sciences. After considering the inclusion criteria, 96 patients including 22 men (23%) and 74 women (77%) between 13 and 70 (mean ± standard deviation=30.9±10.4) years were selected for this study.

The inclusion criteria for the current study were absence of any inflammation, ulcer, pathology, deformity or surgical scars on the lips, no history of physical trauma to the lips, no smoking and no lip chewing habits.

Individuals allergic to cosmetics and subjects with non-Iranian ethnicity were excluded from the study (17,18). The study protocol and objectives were thoroughly explained to the participants and written informed consent was obtained from them. The study was approved by the Ethics Committee of Shahid Beheshti University of Medical Sciences, Dental School (reference # IR. SBMU. RIDS. REC. 1394. 150).

-Recording lip prints

Materials used in order to record lip prints were: black solid, non-glossy, oil-free lipstick (after an experimental lip print recording with different lipsticks, this type was selected for clarity in producing the best lip print and capability of disinfection), 50 mm Scotch tape, plain A4 white paper, make-up remover wipes, povidone-iodine cleansing solution and a pencil sharpener.

The technique of recording was selected according to Costa and Caldas (18). The lips of the subjects were cleaned thoroughly by gently wiping a cotton roll dipped in povidone-iodine cleansing solution before taking the prints. The lipstick was gently applied to the vermilion of both upper and lower lips. After two minutes - while participants were told to put their lips in repose- 50 mm of Scotch tape was pressed gently from the center to the corners of the lips. The participants were asked to refrain from moving their lips during the procedure to avoid any distortions on the print recordings. Scotch tape was then removed from the lips and stuck onto a white paper in order to provide a permanent record, which could be studied at any time. The recording procedure was repeated in case of observing any defects to ensure having a clear record of each participant. Attention was paid to hygiene while recording the lip prints. After confirming the proper registration of all areas of the lips on paper, the lipstick remaining on the lips was cleaned using make-up remover wipes. The tip of the pencil lipstick was then sharpened and disinfected by soaking in povidone-iodine for use by the next participant.

-Analysis of the lip prints

Lip prints were divided into six sextants (three areas in each lip) by drawing two lines, perpendicular to the transverse line, passing the two highest points of the philtrum including right upper lip (RUL), middle upper lip (MUL), left upper lip (LUL), left lower lip (LLL), middle lower lip (MLL) and right lower lip (RLL). The two most lateral parts of the lip prints were excluded from the study as it was usually impossible to register them properly. The obtained prints were examined carefully under a magnifying lens (3X).

**Figure 1:**
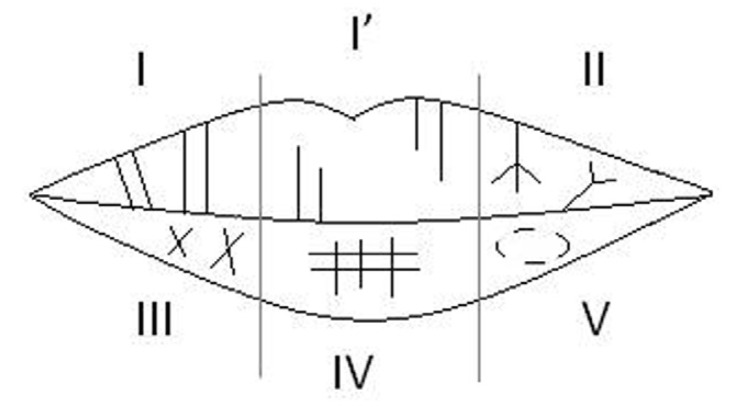
A diagram showing the lip groove types; I: Complete vertical, I’: Incomplete vertical, II: Branched groove, III: Intersected groove, IV: Reticular pattern, V: Irregular morphology.

The analysis of the records was done using the classification proposed by Suzuki and Tsuchihashi (10) (Fig. 1) since it is the most commonly used classification worldwide (7,12,14). The prevailing line pattern of each sextant was reported as its final type. According to Prabhu *et al.*, (17) some modifications were considered including: Bifurcated lines or lines having more than two branches (originating from the same line) were considered as class II. Furthermore, only intersected lines that form an ‘X’ pattern with almost equal lengths of the arms and without any superimposition were considered as class III. Also, lines with multiple interconnections, or areas having lines showing characteristics of various – type I to IV – classes without the possibility of strictly choosing one single type, were considered as class V.

To assess the validity and reliability of the analysis process, each lip record was blindly studied by two trained observers four times in four different days.

To determine the individuality of the lip prints, lips showing the same lip pattern types in the same sextants were differentiated by comparing the lip length, angles and the branching pattern of the grooves.

-Statistical analysis

The data were statistically analyzed using SPSS 18.0 software. The Pearson’s chi-square test was used to assess possible differences between males and females. The level of statistical significance was set at *P* < 0.05.

Also, a weighted kappa test was used to determine both reliability (intra-observer) and validity (inter-observer and comparison of the result of each observer with the final result) of the lip pattern reports by the observers.

## Results

**Figure 2:**
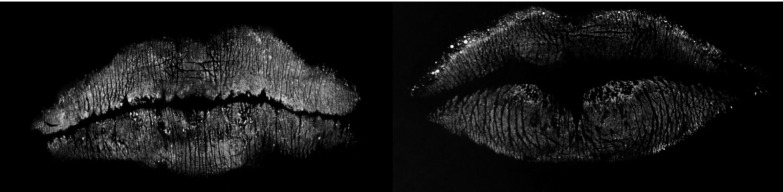
Two cases showing the same lip pattern types in all sextants. However, in the LUL sextants, different characteristics of the grooves were seen.

No exactly identical lip print patterns were observed in the subjects. Specific patterns of branching and location of the lip grooves were evident even in cases showing the same lip pattern types in all the six compartments (Fig. 2).

The distribution of different patterns of lip prints among the studied population is demonstrated in  Table 1. In the current study, the most predominant pattern recorded was type V, which constituted 33.16% of all patterns, followed in order by type I (24.13%), type II (18.75%), type IV (11.63%), type I’ (9.72%) and type III (2.60%). Furthermore, the patterns had different distributions in the sextants. The highest frequencies of type I’ (21.9%), IV (46.9%), V (43.8%), III (7.3%), I (53.1%) and II (44.8%) were in sextants LUL, MUL, RUL, LLL, MLL and RLL, respectively.

**Table 1:**
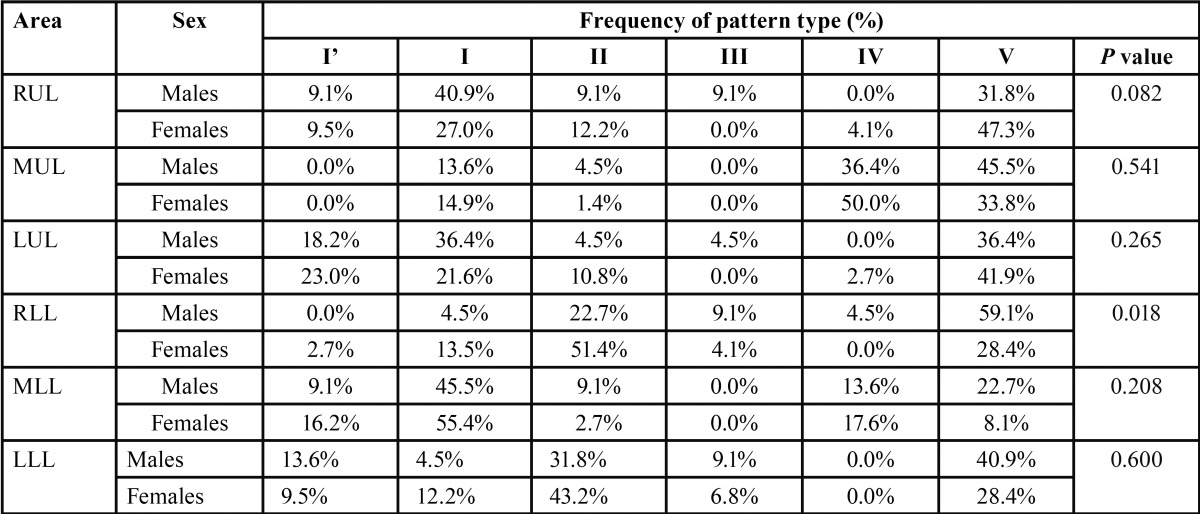
Distribution of lip print patterns based on sex and site (%). Right upper lip (RUL), middle upper lip (MUL), left upper lip (LUL), right lower lip (RLL), middle lower lip (MLL), left lower lip (LLL).

**Figure 3:**
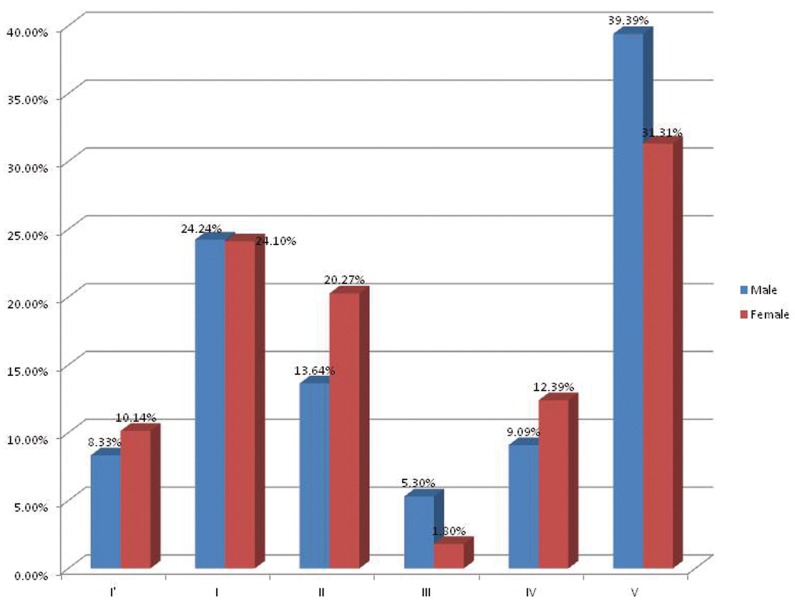
Frequency (%) of lip print patterns in both males and females.

Among males, type V was the most common (39.39%), and type III was the least common (5.30%) pattern. Among females, the same trend was also observed (type V: 31.31%, type III: 1.80%). The distribution of patterns in both males and females is demonstrated in figure 3.

Using the Pearson’s chi-square test, no significant difference was observed between the lip print patterns of males and females in different areas (*P* > 0.05) with the exception of the sextant RLL (*P* = 0.018).

Implementing the weighted kappa test, very good intra-observer reliability and inter-observer validity (weighted kappa > 0.9) were noticed.

## Discussion

It is important to have various methods of identification as alternatives in situations such as crime scenes, accidents and mass disasters (18,19). In this regard, lip prints could be a useful adjunct to fingerprints and teeth for human identification (20,21).

An important aspect affecting lip printing is the method by which the prints are recorded. The status of the lips upon recording is of great importance (19). As suggested by several authors, the closed-mouth position, with the lips in repose, exhibits well-defined grooves suitable for human identification studies (12,22). The amount and uniformity of the lipstick applied to the lips can affect the accuracy of the records as well (17). Thus, in the current study, the lipstick was applied by a trained individual and the subjects were asked not to move or rub their lips during and after applying; thus, the uniformity and even the thickness of the applied lipstick were attempted to be standardized. Moreover, the type of substrate used to capture the prints (plane white paper or cellophane tape) and the pressure and direction by which the substrate is applied may alter the lip prints (17,19). Thus, the method described by Costa and Caldas (18) was selected in this study because of the accuracy of the details captured. Although an effort was made to reduce the inter-operator differences in this study by using the exact same procedure, there is still a need for development of a standard method for recording lip prints.

The type of lipstick used is also important from different aspects. For infection control, the lipstick should be sanitizable after each use. For this purpose, a pencil lipstick was used, which could be soaked in povidone-iodine and the tip could be sharpened after each use. The lipstick should have the potential of providing an even thickness in different regions of the lip and clear imprints of grooves that can be easily studied (18). Last but not least, it should be easy to remove from the lips leaving no trace (17). After trying different types of lipsticks, a dark non-glossy, oil-free pencil lipstick was selected.

We used the classification by Suzuki and Tsuchihashi because it is the most commonly used classification system in the literature (7,12,14,23) and it is easy to use and interpret (24). Lip prints are used in association with critical issues such as human identification and criminal investigations. In this study, the results from the kappa test revealed that the subjective nature of lip print examination does not affect the accuracy of the results and therefore any trained observer can identify lip prints with statistically insignificant errors. Thus, a precise universal approach is needed in this regard.

To the best of our knowledge, this is the first cheiloscopic study carried out in Iran. We found lip print patterns to be unique among the study subjects, which is in agreement with previous studies done on different populations (12,14) such as the one performed by Costa and Caldas (18) that confirmed the ability to discriminate individuals by means of lip prints in a Portuguese population. Domiaty *et al.*, (19) in Saudi Arabia also confirmed the uniqueness of the lip prints even among twins and family members. Ragab *et al.*, (22) also stated the specificity of lip prints in an Egyptian population as no two subjects showed absolutely similar lip print patterns.

The most common pattern was type V and the least common was type III in our study subjects. However, different prevalence of lip print patterns has been reported in different populations. For instance, a study conducted on a Portuguese population reported type II to be the most common and types I’ and V to be the least common types (18).

The current study showed that type V was the most common pattern in both Iranian males and females. However, the sextant in which it was most frequently seen differed between the two sexes (RLL in males, LUL in females). On the other hand, type III was the least common pattern in both sexes and it was seen mostly in LLL. This pattern was not seen at all in the upper lip of females. In the recent years, some authors have claimed differences in lip print patterns of males and females (7,23). For example, Costa and Caldas (18) studied a group of the Portuguese population and reported type III to be the most dominant pattern in males (52%) and type II to be the predominant one in females (44%). Likewise, Singh *et al.* (24) reported that type III was seen most commonly in males (43.3%) while type I was most commonly seen in females (46.7%). In addition, Sharma *et al.* (6) stated that the predominance of patterns in males differed from that in females. However, in their study, no significant difference was found among males and females. Similarly, Ragab *et al.* (22) did not notice any association between lip print pattern distribution and sex. Prabhu *et al.* (17) found equal prevalence of the same patterns in both sexes.

In conclusion, lip printing can be a useful adjunct to fingerprinting for human identification. However, it has some limitations that restrict its unanimous acceptance precluding its use as a legal document in the courts of law. There is a gap of information in this field due to limited studies available in this regard; although lip prints have the potential of being efficient and practical for human identification in forensic medicine. Therefore, there is still a need for further large-scale studies for long-term evaluation of lip prints in order to validate the results and come to a unanimous consensus. Moreover, a commonly accepted universal method of collecting lip prints and interpreting the records should be developed to standardize these findings (i.e. computers and scanners). In addition, as with fingerprints, a worldwide database for lip prints should be established for their large-scale use in criminal investigations and disasters.
